# DLL Design with Wide Input Duty Cycle Range and Low Output Clock Duty Cycle Error

**DOI:** 10.3390/mi16111223

**Published:** 2025-10-27

**Authors:** Binyu Qin, Haoyu Qin, Chenyu Fang, Leilei Zhao, Peter Poechmueller

**Affiliations:** 1 School of Integrated Circuits, Shandong University, Jinan 250101, China; binyu.qin@mail.sdu.edu.cn (B.Q.);; 2 School of Computer Science and Technology, Shandong University of Technology, Zibo 255049, China; 3 School of Information and Control Engineering, Qingdao University of Technology, Qingdao 266520, China

**Keywords:** DRAM, DLL, BBPD, dual loop

## Abstract

This paper presents the design of a Delay-Locked Loop (DLL) with a simple architecture and a wide input clock duty cycle range. The design is tailored to meet the increasing data rate and stringent clock requirements of modern semiconductor chips, with particular applicability to dynamic random-access memory (DRAM) systems. The structure features two Bang-Bang Phase Detectors (BBPDs) to adjust the rising and falling edges of the divided clock. Implemented using a 65 nm CMOS process, the design was verified through simulation. At a working frequency of 3.2 GHz, the input clock duty cycle range spans from 18% to 72%, with a maximum output clock duty cycle error of just 0.6%, a peak-to-peak jitter of 15.73 ps, and a power consumption of 12.7 mW.

## 1. Introduction

In today’s rapidly advancing semiconductor technology field, particularly in high-performance computing, artificial intelligence, and big data analytics, the demand for higher data rates is increasing. This demand is driving continuous innovation and advancement in computer storage technologies. As a core component for data storage and retrieval, the performance of DRAM is crucial to the overall system efficiency and response time. Especially during high-speed data transmission, the precise synchronization between DRAM’s output data, Data Strobe (DQS) signals, and the global clock signal is directly related to the stable access to data and the overall system reliability [1].

With the continuous evolution of semiconductor technology, Double Data Rate 5th Generation (DDR5) has emerged as the next-generation memory standard, achieving data rates of up to 6400 Mbps. This represents a significant bandwidth increase compared to the previous generation DDR4. This breakthrough not only effectively supports larger-scale parallel data processing but also provides more efficient solutions for applications in cloud computing, big data processing, high-performance graphics rendering, and gaming. DDR5’s external input clock frequency reaches 3.2 GHz, providing a solid foundation for such high-speed data transmission. However, with the increase in data rates, the precision requirements for clock synchronization also grow exponentially. To ensure stable system operation and minimize performance fluctuations caused by clock errors, it is necessary to further optimize the generation and transmission of clock signals and to precisely control the timing alignment between the DQS signal and the global clock signal. Therefore, in the design of high-speed DRAM modules, it is essential not only to focus on enhancing data rates but also to comprehensively optimize aspects such as power management, signal integrity, and clock driving, ensuring the system’s reliability and stability at high frequencies.

As DRAM operating frequencies continue to rise and the demand for large-scale, high-quality data processing in artificial intelligence (AI) applications intensifies, the design of DLLs requires in-depth optimization and continuous improvement. Firstly, during high-speed data transmission, it is essential to ensure precise alignment between the clock and data to meet the stringent accuracy requirements of AI for data processing. This involves key technologies such as Clock Data Recovery (CDR) [2]. Secondly, to ensure consistently stable and reliable performance, it is crucial to provide robust data transmission support for AI applications, thereby driving the remarkable development of artificial intelligence under the powerful enabling capabilities of semiconductor technology. This also requires deep consideration of specialized fields such as Signal Integrity (SI) [3].

In the practical application of DRAM, Delay-Locked Loops (DLLs) and Phase-Locked Loops (PLLs) are commonly used to synchronize output data and the DQS signal with the global clock [4]. In Double Data Rate (DDR) Synchronous Dynamic Random-Access Memory (SDRAM), the clock duty cycle is crucial for eye diagram margin and needs to be maintained steadily at approximately 50%. Through the synchronized operation of DLLs and PLLs, timing deviations in data transmission can be minimized, thereby reducing the probability of data errors. A well-maintained clock duty cycle not only ensures sufficient eye diagram margin but also enables the correct recognition of data signals even in the presence of certain interference, thus enhancing system reliability. Compared to PLLs, DLLs do not accumulate jitter [5], and they are less sensitive to process, voltage, and temperature (PVT) variations. Additionally, DLLs feature simpler designs, occupy smaller chip areas, and are more adaptable to future technology upgrades. This makes DLL-based designs more scalable in the context of ongoing technological evolution [1].

**Figure 1:**
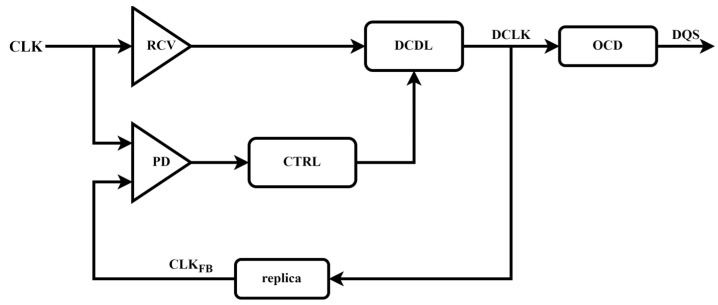
Traditional DLL Structure.

The traditional DLL structure is shown in Figure 1. After the external input clock is processed by receiver (RCV) and Digital—Controlled Delay Line (DCDL), the CLK_FB_ signal is obtained through the replica unit. Subsequently, this signal and CLK are input into the phase detector (PD) for phase—detection operation. The output signal of the phase detector generates a digital control signal through the CTRL module, and this signal acts on the DCDL, aiming to adjust the delay of the clock signal. When the DLL is locked, the output signal DQS of on-chip Driver (OCD) will be aligned with CLK.

As the operating frequency of DRAM continues to increase, the inherent jitter issues in DLLs have become more pronounced, significantly affecting the stability and locking time of the loop. Particularly at high frequencies, the fixed jitter within the DLL can lead to a decrease in clock signal accuracy, thereby affecting the synchronization of data transmission and the overall performance of the system. Additionally, the delay line during operation is influenced by various factors such as signal attenuation, clock skew, and phase noise, which may cause the output clock to suffer from missing or distorted signals. This, in turn, can impair the accuracy of data sampling and reduce the reliability and stability of the system.

To address these issues, the implementation of a Duty Cycle Correction (DCC) Circuit within the DLL is crucial. The DCC can dynamically compensate for distortions in the clock signal by adjusting the duty cycle in real-time, ensuring accurate data sampling during high-speed data transmission and processing. This not only helps to improve the reliability of data transmission but also effectively reduces jitter and uncertainty induced by clock signals, optimizing the quality of the clock signal. Furthermore, the DCC enhances the system’s immunity to interference, ensuring that stable clock signals are provided to each module, thus improving the overall performance and stability of the system. By incorporating this key technology, the system can maintain efficient and reliable operation in complex working environments, meeting the stringent demands for data transmission accuracy and system stability in high-frequency operations.

Traditional Duty Cycle Correction modules have a limited duty cycle adjustment range, and most are implemented in analog form, making them more susceptible to process, voltage, and temperature (PVT) variations. Jincheol Sim applied a Bang-Bang Duty Cycle Detector (BBDCD) to achieve a DCC design with a maximum error of 1.5%, but the adjustable range was limited to 37–63% [6]. Ji-Hoon Lim added a frequency divider in the DLL, successfully achieving duty cycle adjustment between 20 and 80%. However, he still incorporated a DCC structure for duty cycle correction, using multiple logic switching circuits, which increased the complexity of the design. Moreover, the logic switching limited its application in high-frequency circuits [7]. Chien Yu Lin utilized a feedback-based analog DCC to achieve duty cycle adjustment between 20 and 80%, but it remains highly susceptible to PVT variations [8].

This paper presents the design and implementation of a new dual-control loop DLL (DCL-DLL) structure. By incorporating a frequency divider, the external input clock duty cycle range is extended, improving the working frequency and stability of the DLL. We have added a new loop 2 to replace the traditional DCC unit on the basis of the traditional DLL. The delay line DL_2_ is used in the second loop for duty cycle adjustment, eliminating the need for additional duty cycle correction units, thus offering higher robustness. The phase detector employs a BBPD that is insensitive to frequency, enabling phase detection for input clocks at different frequencies. The second part of the paper discusses the current structure of the DLL, providing a detailed description of the architecture of each module. The third part presents the simulation results.

## 2. Structure

Duty cycle mismatch represents a prevalent yet challenging issue that significantly impacts timing accuracy and consequently affects overall system performance. This challenge becomes particularly critical during high-speed data transfer operations, where stringent requirements for timing precision and stability mean even a tiny timing error can lead to data transmission failures.

The proposed DCL-DLL architecture is specifically designed to achieve precise alignment between the rising edges of input clock (CLKT and CLKB) and output clock (DQS), while maintaining an exact 50% duty cycle for DQS (excluding OCD effects). Among them, T and B are only used as markers. Usually, we consider T to be the positive phase and B to be the negative phase. To accomplish this, the design incorporates a secondary control loop that ensures stable and accurate 50% duty cycle regulation for the DCLK clock. Compared to traditional DLL incorporating analog DCC, the DCL-DLL structure exhibits superior architectural simplicity. This streamlined design significantly reduces development effort for complex analog circuits, enhances system stability and reliability, and ensures uncompromised data transmission integrity. In contemporary applications demanding extreme data accuracy, this safeguard is indispensable for normal system operation and effective data utilization.

**Figure 2:**
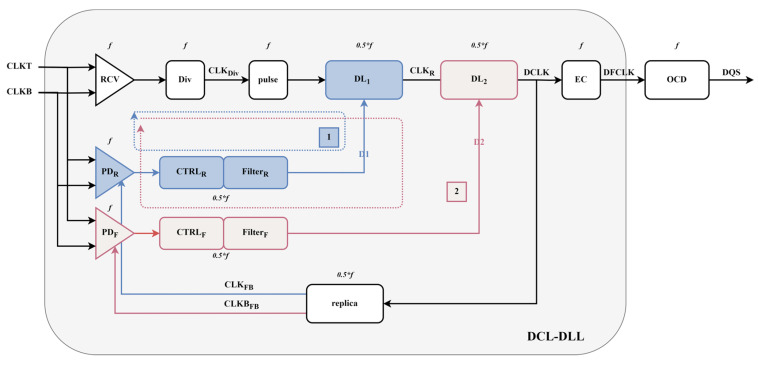
Proposed Dual-Control Loop DLL.

Figure 2 shows the block diagram of the proposed DCL-DLL structure, which consists of several key modules, including a receiver (RCV), a frequency divider (Div), two digital delay adjustment lines (DL_1_ and DL_2_), phase detectors (PD_R_ and PD_F_), controllers (CTRL_R_ and CTRL_F_), and an edge combiner (EC).

The receiver (RCV) is used to receive the external input clock signal. It includes necessary signal buffering and level conversion, providing a reliable clock reference for the DLL. The frequency divider (Div) performs integer division on the received and processed reference clock signal. It aims to strategically reduce the internal operating frequency of the loop, thus effectively improving the noise immunity of subsequent modules and reducing the complexity of their timing design. Due to the subsequent locking strategy, we processed the CLK_Div_ signal and increased its pulse width after passing through the pulse module. The digital delay lines (DL_1_ and DL_2_) are usually composed of a series of digitally controllable basic delay units connected in cascade. They are responsible for dynamically adjusting the number of effective delay stages under the command of the controller to achieve quantitative adjustment of the signal propagation time. The core objective is to precisely align the rising edge of the reference clock (CLKT and CLKB) and the output clock (DQS), and this is often achieved through a two-stage structure design to separate coarse and fine adjustments. The phase detectors (PD_R_ and PD_F_) continuously monitor and quantize the instantaneous phase difference between corresponding edges of the input reference clock and the internal feedback clock in real-time. This phase error is converted into a digital control signal indicating the error polarity. The loop where PD_R_ is located is used for the delay adjustment of the rising edge of DCLK, while the loop where PD_F_ is located is used for the adjustment of the falling edge of DCLK. The controllers (CTRL_R_ and CTRL_F_) are used to receive the phase error information output by the PD. Based on specific digital control algorithms, they generate precise digital control words (D1, D2) to dynamically drive the delay lines (DL_1_ and DL_2_) to adjust the delay increment or decrement, thus achieving precise clock alignment and duty cycle control. The edge combiner (EC) receives the clock signal (DCLK) that has been precisely adjusted by the delay line. It is responsible for synthesizing it into a final clock (DFCLK) with low jitter and a stable target duty cycle, ensuring the output signal frequency. Finally, it outputs the adjusted and optimized signal to the Output Driver (OCD).

This DCL-DLL-based design effectively solves the duty cycle distortion and timing skew issues that traditional DLLs may encounter in high-speed operating environments, providing more stable clock signal support for the system. Through the coordinated operation of these key modules, the DCL-DLL can offer higher precision clock alignment and stronger resistance to interference in complex application scenarios, meeting the stringent clock signal quality requirements of modern high-performance computing and communication systems.

After the DLL is initialized, Loop 1 begins operation. The external differential clocks CLKT and CLKB are divided by the receiver (RCV) to generate the signal CLK_Div_. CLK_Div_, after passing through the delay line, generates the clock signal CLK_F_, which is then used in the replication module to produce the differential signals CLK_FB_ and CLKB_FB_. These signals are then phase-detected with the external clock signal. Since the frequencies of the external input clock CLKT and the feedback clock CLK_FB_ are different, we use the sampling BBPD as our phase detector. Based on the proposed BBPD structure, it is necessary to conduct a phase comparison between the differential input clock (CLKT and CLKB) and the feedback clock CLK_FB_. When CLKT leads CLK_FB_, the Bang-Bang Phase Detector-Rising (BBPD_R_) outputs “1”; otherwise, it outputs “0”. The output of BBPD_R_ is processed to control DL_1_, aligning the rising edge of CLK_FB_ with the rising edge of CLKT. When the rising edges of CLK_FB_ and CLKT are aligned, the Lock_R_ signal is set to “1”. At this point, Loop 2 begins operation. When CLKT leads CLKB_FB_, the BBPD_F_ outputs “1”; otherwise, it outputs “0”. The output signal D2 of BBPD_F_ controls DL_2_, ensuring the rising edge of CLKB_FB_ aligns with the rising edge of CLKT. The rising edges of the differential clocks CLK_FB_ and CLKB_FB_ correspond to the odd and even rising edges of CLKT, respectively, thus enabling duty cycle adjustment of the CLK_F_ signal. The CLK_F_ signal is then frequency-multiplied to produce the DQS signal, which is synchronized with CLKT and has aligned rising edges. Any delay introduced during the frequency multiplication process is compensated for in the replica module. Compared to the fixed 0.5*T delay line used in the circuit to adjust the duty cycle, the feedback duty cycle adjustment circuit in the loop offers a wider adjustable duty cycle range and is not affected by output buffer delays [7].

**Figure 3:**
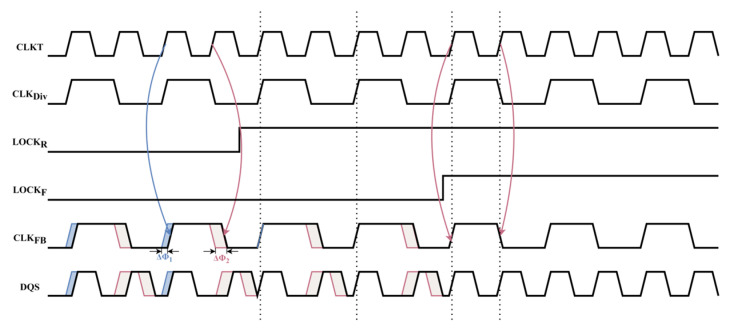
Timing diagram of DCL-DLL adjustment.

In the designed dual-loop system, Loop 1 is primarily responsible for eliminating the phase error ΔΦ_1_, as shown in Figure 3. Specifically, the delay adjustment in Loop 1 ensures that at the end of the loop, the rising edge of the feedback clock (CLK_FB_) is precisely aligned with the rising edge of the input clock (CLKT), thus ensuring clock signal synchronization. Once this alignment is completed, the LOCK_R_ flag is raised, indicating that Loop 1 has successfully locked and stabilized. Loop 2 is then used to further eliminate the existing phase error ΔΦ_2_, ensuring that after adjustment, half of the period of the feedback clock (CLK_FB_) exactly matches the period of the input clock (CLKT). This way, Loop 2 ensures precise synchronization of the clock signal, preventing data transmission errors or timing instability caused by phase differences.

During dynamic adjustment, the operating frequency of the dual-loop system is reduced by half. This design enables the system to operate at a lower frequency, thus enhancing its resistance to high-frequency noise and interference. Compared to high-frequency signals, low-frequency signals are less sensitive to noise. Therefore, by lowering the frequency, the dual-loop system can effectively increase the system’s immunity to interference, ensuring high-precision clock signal output even in complex environments. This dual-loop structure not only optimizes the system’s clock synchronization performance but also enhances its robustness in dynamic environments, ensuring the stability and reliability of the system under various complex conditions. In contrast to another DLL design with dual delay lines [9], the single delay line structure in this design avoids the matching issue of two delay lines in the dual delay line structure. Additionally, since no multiplexers are used, loop switching is not required, which improves the theoretical maximum operating frequency.

## 3. Detailed Composition of the Modules

To significantly increase the duty cycle adjustment range of the DLL, the DCL—DLL adds a frequency divider into loop structure. The direct impact of this crucial measure is that the frequency of the clock DCLK after delay adjustment becomes half of the frequencies of the external input clocks (CLKT and CLKB). It should be noted that the frequency of the output clock CLK_FB_ processed by the replica module is not the same as that of CLKT. In view of this special situation, the adopted BBPD must have the ability to accurately output the phase error information when there are differences in the input clock frequencies. And within each clock cycle of CLK_FB_, this BBPD can only output the phase error result once, to ensure the accuracy and stability of the system.

**Figure 4:**
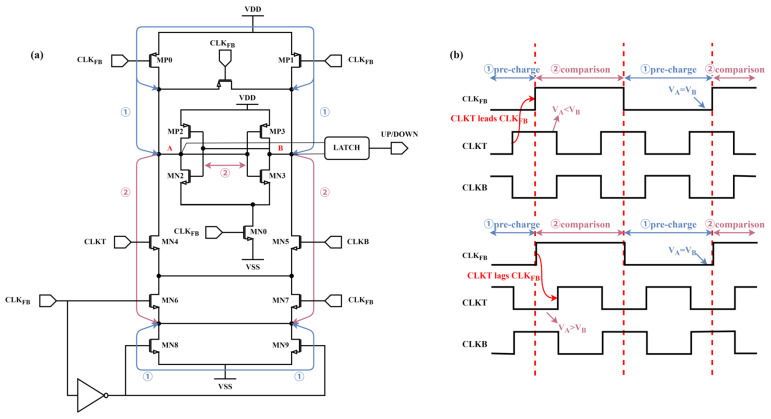
Block diagram of BBPD: (**a**) Schematic diagram of BBPD; (**b**) BBPD work timing diagram.

Figure 4a shows the block diagram of the Phase Detector. Due to the difficulty in replicating the delay of the RCV, the proposed DCL-DLL structure performs phase detection between CLKT and CLK_FB_, even though these two signals have different frequencies. Therefore, the BBPD used is frequency-insensitive and can function properly even with differing input clock frequencies. The BBPD phase detector operates in two states: the “pre-charge” state and the “comparison” state.

The working timing diagram of BBPD is shown in Figure 4b. When CLK_FB_ is “0,” the BBPD operates in the “pre-charge” phase, and both points A and B charge up to VDD. MN8 and MN9 are turned on, and their drain terminals are set to “0.” When CLK_FB_ transitions from “0” to “1,” if at this time, CLKT is at a high level (CLKB is at a low level), then MN0 will be turned on and discharge point A, causing the potential at point A to be pulled low. Through positive feedback, point A is set to “0” and point B is set to “1”. This state will be saved and output by the latch. And a high level of CLKT means that the phase of CLKT is ahead of CLK_FB_. When CLK_FB_ changes from “1” to “0”, points A and B are recharged to a high level. For the latch, it will maintain the result of the previous comparison. Therefore, in one CLK_FB_ clock cycle, BBPD will perform a phase comparison. Similarly, when CLKT lags behind CLK_FB_, at the start of “comparison”, the potential at point B will be pulled low, resulting in the opposite output. This is the “comparison” phase. The function of the Phase Detector can be seen as sampling CLKT with respect to CLK_FB_. When CLKT leads CLK_FB_, the output signal “up” is set to “1.” When CLKT lags behind CLK_FB_, the output signal “down” is set to “1.”

**Figure 5:**
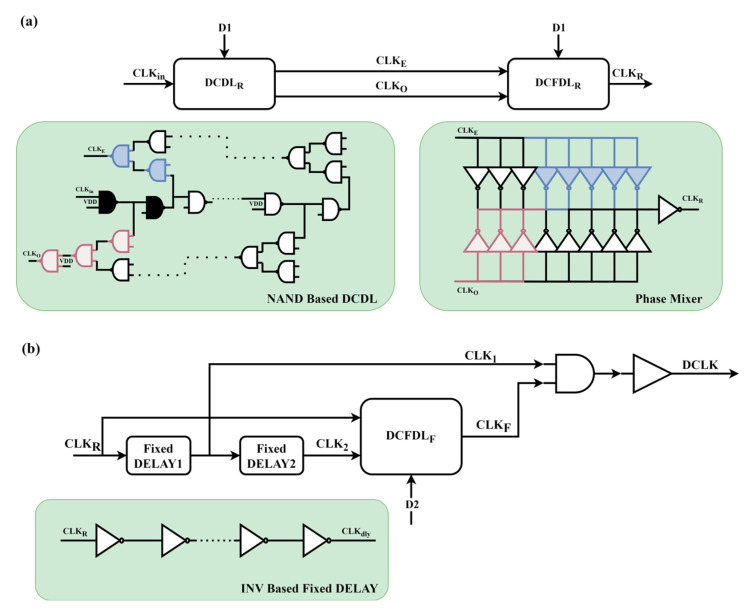
Block diagram of the delay lines in the proposed DLL: (**a**) delay line DL_1_; (**b**) delay line DL_2_.

In Loop 1, the delay line DL_1_, as shown in Figure 5a, employs a combination of coarse and fine adjustment techniques to achieve high-precision clock signal adjustment. Specifically, the Digital control Coarse Delay Line (DCDL_R_) consists of multiple NAND delay cells. The number of NAND delay cells through which the clock signal (CLK_in_) passes is controlled by digital encoding, allowing for the initial delay adjustment of the clock signal. The delay generated by this process is N* 2 * T_dly_, where T_dly_ is the delay of a single NAND gate, N is an integer and is controlled by the digital code D1. The adjusted clock signals, CLK_E_ and CLK_O_, are then input into the Digital Control Fine Delay Line (DCFDL_R_). The fine adjustment module further adjusts the signal using digital encoding to perform interpolation, resulting in a highly accurate clock signal output, denoted as CLK_R_.

Phase interpolation plays a crucial role in this process, significantly enhancing the precision, stability, and synchronization of the clock signal. By interpolating between multiple delay cells, the system can achieve higher clock adjustment resolution. Compared to traditional clock adjustment methods, phase interpolation effectively reduces jitter and timing errors in the clock signal, ensuring more precise data transmission synchronization. This fine clock control not only optimizes the clock signal synchronization in the data transmission process but also reduces errors caused by clock instability, thereby improving the reliability of data transmission and the overall performance of the system. In addition to precise clock synchronization and error reduction, phase interpolation also contributes positively to increasing the system’s bandwidth and data rate. As data transmission rates continue to rise, the precision of clock signals becomes even more critical. Phase interpolation enables the system to handle higher data rates while maintaining clock signal stability, thereby meeting the demands of high bandwidth. This is especially important for modern high-performance computing and communication systems, where the quality of the clock signal directly impacts the accuracy of data transmission in high-speed signal transmission scenarios.

The delay line DL_2_ of Loop 2, as shown in Figure 5b, consists of two fixed delay units (FDL_1_ and FDL_2_) and another DCFDL_F_ module. The phase mixer outputs the clock signal CLK_F_. The rising edge of CLK_1_ is determined by D1. When Loop 1 is locked, the rising edge of CLK_1_ is fixed (assuming that the subsequent D1 does not change temporarily). Therefore, the information from Loop 1 will be transmitted to DCLK fixedly, even if Loop 2 is changing at this time.

**Figure 6:**
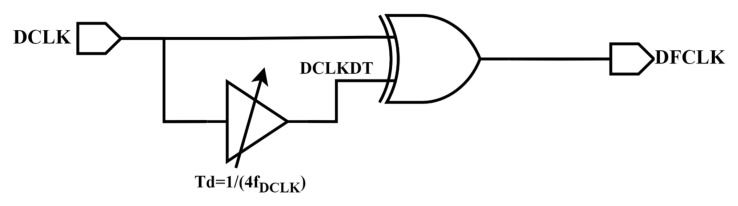
Edge combiner.

The edge combiner structure is shown in Figure 6, and this structure is a simple frequency multiplier. For the clock DCLK, delay it by 1/4 of a clock cycle to obtain the clock DCLKDT. Then, performing an exclusive OR operation between DCLK and DCLKDT, the frequency doubling function of the DCLK clock can be achieved.

## 4. Simulation Results

The proposed DCL-DLL is designed using a 65 nm CMOS process. With a 1.2 V supply voltage and 1.6 GHz/3.2 GHz operating frequency, the total power consumption is around 6.7 mW (6.7 GHz) and 12.7 mW (3.2 GHz), with the delay lines DL_1_ and DL_2_ being the primary sources of power consumption. At 1.6 GHz (tt corner), the allowable input duty cycle range is approximately 14% to 88%, and the measured peak-to-peak jitter is about 22 ps. At 3.2 GHz (tt corner), the allowable input duty cycle range is approximately 18% to 72%, and the measured peak-to-peak jitter is about 15.73 ps. In simulations with varying input duty cycles, the peak-to-peak jitter remains relatively constant, with the jitter error primarily attributed to the phase detection accuracy of the BBPD and the interpolation accuracy of the DCFDL.

**Figure 7:**
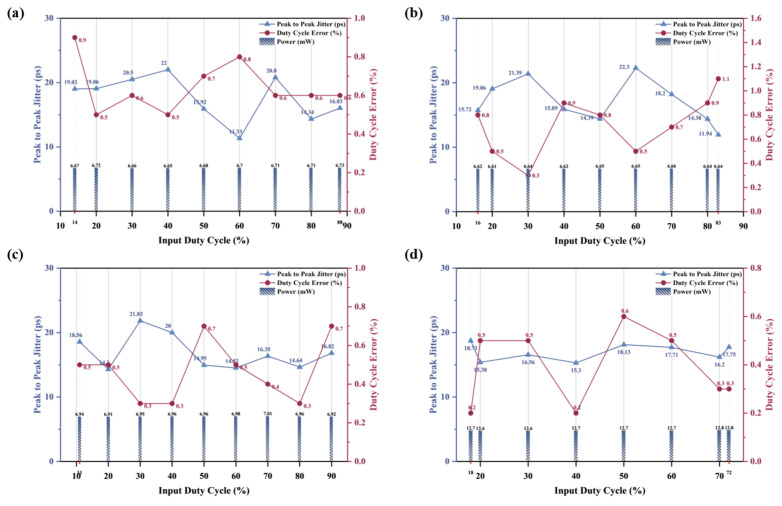
Simulation results for input clocks at 25 °C with different duty cycles: (**a**) 1.6 GHz, 25°, tt corner; (**b**) 1.6 GHz, 25°, ss corner; (**c**) 1.6 GHz, 25°, ff corner; (**d**) 3.2 GHz, 25°, tt corner.

For external input clocks with different duty cycles, the peak-to-peak jitter of the output clock after the current DLL locks is less than 22.3 ps. By simulating different corners of the DCL-DLL at 25 °C, the results are shown in Figure 7. At the tt corner, for an input clock with a duty cycle of 14–88%, the duty cycle error of the generated DQS clock by the DCL-DLL is 0.5–0.9%. Compared with the input clock CLK, the peak-to-peak jitter of the generated DQS clock is approximately 11.33–22 ps. When the input clock duty cycle is 50%, the average power consumption of the DCL-DLL is about 6.68 mW. At the ss corner, for an input clock with a duty cycle of 16–83%, the duty cycle error of the generated DQS clock by the DCL-DLL is 0.3–1.1%. Compared with the input clock CLK, the peak-to-peak jitter of the generated DQS clock is approximately 11.94–22.3 ps. When the input clock duty cycle is 50%, the average power consumption of the DCL-DLL is about 6.65 mW. At the ff corner, for an input clock with a duty cycle of 11–90%, the duty cycle error of the generated DQS clock by the DCL-DLL is 0.3–0.7%. Compared with the input clock CLK, the peak-to-peak jitter of the generated DQS clock is approximately 11.2–21.83 ps. When the input clock duty cycle is 50%, the average power consumption of the DCL-DLL is about 6.96 mW.

We conducted simulations on the DCL-DLL at an operating frequency of 3.2 GHz. In Figure 7d, the adjustable duty cycle input range of the DCL-DLL is 18–72%, and the maximum duty cycle error of the output clock DQS is 0.6%. Compared with the input clock CLK, the peak-to-peak jitter of the generated DQS clock is approximately 12.3–15.73 ps. When the input clock duty cycle is 50%, the average power consumption of the DCL-DLL is about 12.7 mW. When the duty cycle of the external input clock is outside the adjustable range, due to the setup time of the Flip-Flop and the limitation of the effective adjustment bits in Loop 2, situations where the clock cannot be transmitted to the adjustment loop and the loop cannot be locked will occur.

By comparing with Table 1, the proposed DCL-DLL structure has advantages in expanding the duty cycle adjustment range and adjusting high-frequency clocks.

## Figures and Tables

**Table 1:** Comparison with previous works.

	This Work	VLSI, 2021 [10]	TCAS-Ⅱ, 2016 [7]	ISCAS, 2021 [6]
ARCHITECTURE	Dvider + DCL + BBPD	ADDLL	Divider + DCC	BBDCD
Supply Voltage	1.2 V	1 V	1.2 V	1 V
Input Frequency Range	1.6 G/3.2 G	0.1–2.7 G	1.6 G/2 G	1 G–3.2 G
Input Clock Duty Cycle Range	14–88%/18–72%	N/A	19.9–80.4%	37–63%
Peak to Peak Jitter	22 ps/15.73 ps (tt)	5 ps	14 ps	12 ps
AREA	N/A	0.089 mm^2^	0.099 mm^2^	0.001 mm^2^
Power@Fmax	6.68 mW/12.7 mW	49.4 mW	6.6 mW	1.92 mW
Technology	65 nm	90 nm	65 nm	28 nm

## Data Availability

The original contributions presented in the study are included in the article, further inquiries can be directed to the corresponding author.
